# On the primacy and irreducible nature of first-person versus third-person information

**DOI:** 10.12688/f1000research.10752.3

**Published:** 2017-05-31

**Authors:** Patrizio E. Tressoldi, Enrico Facco, Daniela Lucangeli

**Affiliations:** 1 Dipartimento di Psicologia Generale, Università di Padova, Padova, Italy; 2 Studium Patavinum, Università di Padova, Padova, Italy; 3 Dipartimento di Psicologia dello Sviluppo e della Socializzazione, Università di Padova, Padova, Italy

**Keywords:** first-person, third-person, consciousness, mind-brain relationship

## Abstract

In this essay, we will support the claim that at the current level of scientific advancement a) some first-person accounts cannot be reduced to their third-person neural and psychophysiological correlates and b) that these first-person accounts are the only information to reckon when it is necessary to analyse qualia contents.

Consequently, for many phenomena, first-person accounts are the only reliable source of information available and the knowledge of their neural and psychophysical correlates don’t offer any additional information about them.

## Introduction

First-person accounts (1PAs) are phenomenological subjective mental content the person is aware of and that can be communicate to others, if requested or desired, by written, verbal or intentional (conscious) behaviour, e.g. sign language. “ *I feel happy today*”; “ *I see a pink rose*”; “ *This panorama is awesome*”; and “ *I think I had better do it tomorrow*”, etc. are some typical examples. These contents are also defined as “qualia” ( Tye, 2016).

On the contrary, third-person accounts (3PAs), are identical types of accounts plus their neuro and psychophysiological correlates, obtained by people who observe or measure other behaviour and mental contents and processes. “ *He seems happy*”; “ *She’s looking at a rose*”, and “ *He pushed the red button*”, are example of verbal accounts. “ *The power of his EEG alpha band had an increase of 10%, when he relaxed*”; “ *The medial frontal cortex increased its activity when she smiled at her partner*”, and “ *Her heart rate decreased from 80 bpm to 60 bpm, when she heard pleasant music*”, are examples of neuro and psychophysiological correlates of mental activity of the observed person.

In this essay, we will support the claim that a) some 1PAs cannot be reduced to third-person neural and psychophysiological correlates accounts (3PAs). We will not enter here in the debate about how 1PAs can also be considered 3PAs ( Piccinini, 2010) with particular reference to the heterophenomenology as defined by Dennett (2003) and b) that their contents are the only information to reckon when it is necessary to analyse qualia contents, that is, emotions, beliefs, reality interpretations, quality of life and health and their effects on behaviour and the brain activity. Consequently, c) even a complete description of the brain and psychophysiological correlates of these 1PAs does not add any further information about their contents and characteristics.

This approach is at odds with the view that given the subjective and introspective nature of 1PAs, they lack objective contents and hence 3PAs are undeniably more informative.

There is not space here to describe the historical reasons of why, in psychology, 1PAs lost their importance in comparison to 3PAs. For those readers interested in this topic we suggest to refer to Klein (2015b).

## When first-person accounts are the only valid information to consider

Below is a (non-exhaustive) list of phenomena and conditions that can be described and known only by 1PAs whereas the third-person correlates are irrelevant in order to understand their characteristics. For each of the selected phenomena we will present some examples of 1PAs and 3PAs to make evident the different informational value of these accounts as supportive of our main thesis.

### Sensations and Emotions

**Figure 1.**
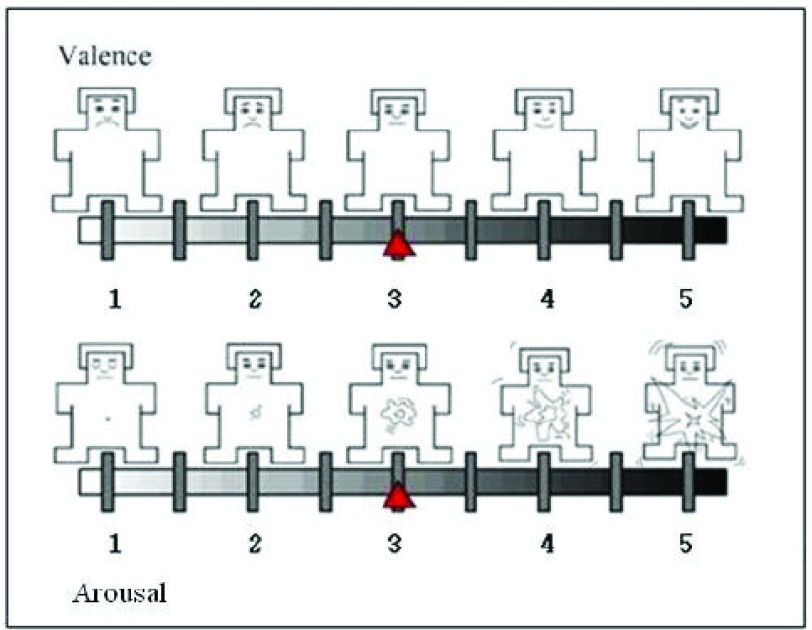
An example of the Self-Assessment Manikin for the measure of emotions’ characteristics. In this case, the participant is requested to rate the emotional valence and arousal of a stimulus on a 5-point scale. This figure has been reproduced with permission from Li *et al.*, 2011.

***Emotions and Emotion (Mood) Disorders*.** Emotions identification and their valence and arousal can be measured only taking in account 1PAs. For example, the Self-Assessment Manikin in different version, see Figure 1 as an example, was used for the database of the International Affective Pictures System, whereas bipolar semantic slider scales from from 1 to 9, were used for the Nencki Affective Picture System ( Marchewka *et al.*, 2014)

As for the measure of emotions triggered by pictures, faces, persons, etc., even the measure of the mood and its disorders can only be done by referring to 1PAs, usually by way of structured questionnaires, e.g. the Beck Depression Inventory ( Beck *et al.*, 1996) or interviews, e.g. The Structured Clinical Interview for DSM-5 (SCID-5; First *et al.*, 2015), in which participants respond with their extent of agreement with statements such as *“I feel sad”* or *“I don’t cry any more than usual”,* etc.

On the contrary, neuro and psychophysiological accounts (e.g. Allen *et al.*, 2004; Lin *et al.*, 2010), consists of biological signals that cannot convey any subjective and qualitative information about their contents but simply represent a correlation with a different type of information. For example, Matsubara *et al.* (2016), found that the anterior cingulate cortex volume could be a distinct endophenotype of bipolar disorders, while the insular volume could be a shared bipolar disorders and major depressive disorder endophenotype. Moreover, the insula could be associated with cognitive decline and poor outcome in bipolar disorders. Can we use this information to integrate our knowledge about the characteristics of bipolar and depressive disorders of those participants?

### Pain

**Figure 2.**
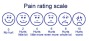
Example of a visual analog scale to measure pain. Participants are requested to rate their perceived pain choosing one of the six different options. No copyrighted figure.

Visual analogue, numerical rating and verbal rating scales (see Figure 2) are commonly used to assess pain intensity in clinical trials and in other types of studies. Among the multidimensional questionnaires designed to assess pain, the McGill Pain Questionnaire and Brief Pain Inventory are valid in many multilingual versions ( Caraceni *et al.*, 2002).

An example of a 3PAs account is “ *The insula ipsilateral to the site of needling was activated to a greater extent during real acupuncture than during the placebo intervention*” ( Pariente *et al.*, 2005). It seems clear that this type of information cannot convey any useful information about the subjective quality of pain of the persons experiencing it.

Another example is offered by Hui *et al.* (2007), who investigated the effects of acupuncture stimulation in eliciting “deqi”, a composite of sensations interpreted as the flow of qi or ‘vital energy’ according to the traditional Chinese medicine. Their procedure was entirely based on 1PAs and described as follows :” At the end of each tactile stimulation or acupuncture procedure, the subject was questioned by another researcher in the team if each of the deqi sensations (aching, pressure, soreness, heaviness, fullness, warmth, cooling, numbness, tingling, dull pain), sharp pain or any other sensations occurred during the stimulation, and to rate its intensity on the scale of 1–10 (1–3 mild, 4–6 moderate, 7–9 strong, 10 unbearable)” page 4.

### Anomalous or non-ordinary experiences

Anomalous or non-ordinary experiences comprise a large group of personal experiences characterized by the lack of any clinical psychopathological syndrome, even if they may appear associated with some of them ( Cardeña & Facco, 2015; Cardeña *et al.*, 2014).

Among these experiences there are:

***Spiritual experiences*.** Spiritual experiences, independently from how they are obtained, e.g. spiritual practices, e.g. meditation ( Chen *et al.*, 2011), spontaneously or by using psychotropic drugs like the psilocybin ( Griffiths *et al.*, 2008), are only based on 1PAs.

The Revised Mystical Experience Questionnaire ( Barrett *et al.*, 2015) is one of the available questionnaires for the investigation of these experiences. Participants are requested to express their degree of experience related, for example to: loss of usual sense of time; experience of amazement; sense that the experience cannot be described adequately in words; gain of insightful knowledge experienced at an intuitive level, etc.

Beauregard & Paquette (2006), investigated the neural correlates of such a type of experiences in a group of Carmelite nuns and found that this state was associated with significant loci of activation in the right medial orbitofrontal cortex, right middle temporal cortex, right inferior and superior parietal lobules, right caudate, left medial prefrontal cortex, left anterior cingulate cortex, left inferior parietal lobule, left insula, left caudate, and left brainstem. Can we achieve better understanding of the quality of these experiences with this information?

***Near-Death-Experiences*.** Near-Death-Experiences (NDEs) are peculiar mental experiences reported by persons who suffered severe injuries, e.g. cardiac arrest ( Agrillo, 2011; Facco & Agrillo, 2012; van Lommel, 2011), characterized by increased vividness and sense of reality with respect to the normal awake state when neither consciousness nor cortical activity is expected: e.g. “Super awake. I could sense things more than I do in my usual state of awareness”, plus other peculiar experiences, for example encounters with spiritual beings: e.g. “I do remember a being of light, God, standing near me” and experiences of living a timeless dimension: e.g. “I became time and space”, etc. (Excerpts from the http://www.nderf.org/Archives/exceptional.html database)

Mobbs & Watt, (2011) are among those who are trying to explain these experiences as simply epiphenomena of some neural activity. For example, they stated: “ *the vivid pleasure frequently experienced in near-death experiences may be the result of fear-elicited opioid release, while the life review and REM components of the near-death experience could be attributed to the action of the locus coeruleus- noradrenaline system”*(page 449). However, statements like these, take for granted that the neural correlates “translate” into subjective experiences forgetting to offer a testable hypothesis on how this transformation can take place. Furthermore, this hypothesis of opioids has several weaknesses ( Ersek *et al.*, 2004; Facco, 2010; Facco & Agrillo, 2012; Lawlor & Bruera, 2002; Vella-Brincat & Macleod, 2007), that is: a) opioids are only weak hallucinogens, b) people administered opioids for pain therapy do not experience NDEs, while their adverse events may include a delirium, the phenomenology of which is totally different with from NDEs; c) No hallucinogens induce standard reproducible experiences, which largely depend on subjects’ personalities, aims of their intake, context and rituality. In other words, when new facts challenge the endorsed axioms and theories, they are first interpreted trying to constraint them within the available knowledge, while their explanation may call for new, yet unknown, laws of nature (i.e., properties of consciousness).

## Memory

Differently from implicit memory, e.g. procedural and associative memory, all aspects of explicit memory, e.g. autobiographical, semantic, have to rely only on 1PAs ( Wilson, 2002). For example, testing autobiographical memory requires the participants to retrieve and describe personal life episodes, e.g. celebrations, diseases, special encounters with friends and relatives, etc.

Moreover Klein (2015a) extensively discussed that in order to qualify as memory, “ *the product of learning needs to be a mental state that includes the feeling that one is reliving a past experience—that is, it provides a directly-given, non-inferential sense that one’s current mental state reflects a happening from one’s past*.” (page 2). This distinction allows to interpret a series of impairments characterized by a dissociation between memory contents and the feeling of ownership of them ( Klein, 2015a)

As to an example of 3PAs, Conway *et al.* (2001), recording the slow cortical potentials, found that left frontal negativity primarily reflects cortical activation associated with the operation of a complex retrieval process, whereas the later temporal and occipital negativity (the result of the retrieval process) reflects activation corresponding to the formation and maintenance of a detailed memory. Can you extract useful information related to the contents and the subjective experience of memory of participants from these data?

## Reasoning

Among the many tasks that can be used to investigate reasoning, one is to judge whether the final statement after a series of propositions is true or false. For example, “All men are animals. All animals are mortal. Hence, all men are mortal.”: True or False?. Papageorgiou *et al.* (2016), investigated the EEG correlates of a series of valid and paradoxical statements and found that “ *During the processing of paradoxes, results demonstrated a more positive event-related potential deflection (P300) across frontal regions, whereas processing of valid statements was associated with noticeable P300 amplitudes across parieto-occipital regions*”. Is there any useful information in these data that can integrate what the participants experience as thoughts, feelings and emotions?

Furthermore, any judgement in terms of true vs false, is closely dependent on culture and available knowledge and, thus, is intrinsically weak and provisional. Judgements on both truth and falsity as well as paradoxes may change over time: for example, the unity of space-time and matter-energy, the Heisenberg’s principle of indetermination and the concept of entanglement look to be true in quantum physics, false or ununderstandable according to classical Newtonian physics. Thus, neurophysiological data about judgements can only provide an estimation of brain mechanisms and, at best, helping one to check whether the subject is processing them as paradoxes or valid statements, without any possible inference on subject’s experience, cultural components and, last but not least, on knowledge and comprehension of the truth, which remains in the realm of mind.

## Beliefs and Self-evaluations

### Beliefs and delusional beliefs

All cultural, ethical, religious, cultural and scientific beliefs as well as all kinds of delusional beliefs, can only be known by using 1PAs (e.g. Coltheart *et al.*, 2011; Jonas & Fischer, 2006; Zeidler *et al.*, 2002).

For example, Kapogiannis *et al.* (2009), investigated the neural correlates of three psychological dimensions of religious belief (God’s perceived level of involvement, God’s perceived emotion, and doctrinal/experiential religious knowledge). Participants 1PAs were obtained by requesting to rate different statements, e.g. *“God cares about the worlds’ welfare”; “All religions have truth”,* on a 7-point Likert scale. The neural correlates of these dimensions were investigated by using fMRI. These authors found different neural networks associated with the three religious beliefs, e.g. more activation of bilateral inferior frontal gyrus, pars triangularis and Brodmann area 45 in relationship with God’s lack of involvement and more activation of the right middle frontal gyrus and Brodmann area 11 in relationship to statements reflecting God’s love etc.

How much information can we add to what we obtained from 1PAs by using these 3PAs?

### Hallucinations

Visual and auditory hallucinations such as hearing voices ( Holt & Tickle, 2014), can be identified and assessed by using 1PAs ( Haddock *et al.*, 1999).

Barkus *et al.* (2007), investigating the neural correlates of non-clinical auditory hallucinations of a group of participants by using the fMRI, found increased activation in the superior and middle temporal cortex. Does this information help to increase what authors already know about the auditory hallucinations of their participants?

### Placebo

The core components of placebo and nocebo effects are expectations/beliefs and conditioned reactions ( Price *et al.*, 2008; Rief & Petrie, 2016). Whereas conditioned reactions can be activated bypassing any mental activity, expectations and beliefs are intrinsically 1PAs independently from whether people are aware or not of them ( Jensen *et al.*, 2012) and cannot be interpretable by using their neural correlates.

### Risk perception

Risk perception both for natural, economic, political and hazard events is another important mental content that can only be measured by using 1PAs ( Sjoberg, 2000).

For example, Schmälzle *et al.* (2011), investigated the HIV risk perception by presenting photographs of unknown persons and recording the EEG evoked response potentials.

They found that the implicit processing of individuals prone to risky behaviour was associated with an early occipital negativity between 240 and 300 ms and a subsequent central positivity between 430 and 530 ms, compared to individuals with safer practices. It appears evident that this information cannot be used to increase the knowledge about risk perception obtained by 1PAs.

### Aesthetic appreciation and judgments

All natural ( Daniel & Meitner, 2001), human ( Berggren *et al.*, 2010), animal and aesthetic appreciation and judgments, can only be assessed by 1PAs ( Leder *et al.*, 2004).

Thakral *et al.* (2012), investigated the neural correlates of van Gogh paintings evoking a range of motion experience by using the fMRI and found that the sensory motion processing region MT+ activity was correlated to the degree of motion experience (but not the experience of pleasantness), whereas the experience of pleasantness (but not motion experience) was associated with an increased activity in the right anterior prefrontal cortex. Can this neural information add any useful information about pleasantness and motion appreciation experienced by these participants?

### Quality of life and health

The World Health Organisation (WHO) define quality of life (QoL) as “ *individuals’ perception of their position in life in the context of the culture and value systems in which they live and in relation to their goals, expectations, standards and concerns*” ( WHO, 1998). QoL is evaluated by different versions of questionnaire of which the best known are those developed by the WHOQOL groups ( WHO, 1998; WHOQOL Group, 1995).

Urry *et al.* (2004) requested their participants to complete self-report measures of eudaimonic (leading a virtuous life and doing what is worth doing) well-being, hedonic well-being, and positive affect and subsequently recorded their EEG activity. They found a greater left than right superior frontal activation association with higher levels of both forms of well-being. May we use this information to gather more details about what already participants reported in their 1PAs?

## First-person accounts are not always reliable

Since the seminal paper of Nisbett & Wilson (1977) evidence has been accumulated showing that people 1PAs can fail in the detection of their decision processes (but see Petitmengin *et al.*, 2013, for a manipulation which reverted the accuracy to a high level).

According to Schooler (2015), 1PAs become unreliable when translation dissociations occur. Translation dissociations “ *correspond to situations in which, while in the process of re-reepresentation, one omits, distorts, or otherwise misrepresents one’s mental state to oneself and/or others.*” page 9.

A typical example is the monitoring of mind-wandering which is typically measured using self-catching and experience sampling techniques. Self-catching asks participants to monitor their mental activity and signal, for example by pressing a button, when they notice their mind activity was off-task. With experience sampling techniques, participants are probed to notice whether their mind was wandering at random time intervals.

Similarly, responses to all interview or to more or less structured instruments for the assessment of 1PAs, can be distorted intentionally or unintentionally for example biased by social desirability ( Huang *et al.*, 1998; van den Noort *et al.*, 2017).

However, these arguments do not confute the main thesis of this essay, that is that 3PAs cannot offer a better information than those obtained by the 1PAs. If we observe that our instruments and procedures used for the knowledge of 1PAs, show some limitations, we can only improve them (see for example Lange, 2017; Pastore *et al.*, 2017).

## Discussion

As anticipated in the introduction, the aim of this essay was that of supporting the claim that at the current level of scientific advancement, there are many varieties of 1PAs whose contents and characteristics can be known and investigated only by these accounts and cannot be integrated with information gathered by 3PAs in particular those related to their neural or psychophysiological correlates.

We have listed ten types of phenomena that can be studied only by referring to 1PAs, even if for each of them there is a legitimate interest in knowing their neural and psychophysiological correlates. However, it is important to realize, on the part of both researchers and the funders of their investigations, that the knowledge of their neural and psychophysiological correlates has nothing to add to the knowledge of these phenomena.

In Table 1 we summarize the characteristics of 1PAs and 3PAs in order to facilitate the understanding of their different nature and hence the irreducibility of 1PAs information to 3PAs ones

**Table 1.** characteristics of 1PAs and 3PAs.

1PAs	3PAs
Subjective phenomenological sensations, feelings, emotions, perceptions, desires, goals, thoughts, etc.	Observations, emotions, feelings related to 1PAs expressions
	Physiological correlates (metabolic, electromagnetic, mechanical) related to 1PAs

Our statement that 1PAs are irreducible to 3PAs, could be falsified by the evidence that it is possible to determine precisely not only the changes but also the qualities of 1PAs only by observing the effects of the interventions on their biological correlates. For example, Saitoh *et al.* (2007) were successful in reducing pain due to spinal cord or peripheral lesions by applying high-frequency repetitive transcranial magnetic stimulation on the primary motor cortex. However, the modification of primary cortex activity didn’t give any useful information about the participants’ change in pain perception. In fact, this information was obtained by asking the participants to rate their pain with a visual analogue scale similar to that presented in Figure 2 and the Short-Form of the McGill Pain Questionnaire.

Pain reduction can also be obtained by acting on mental beliefs and contents. For example, hypnosis may yield a significant increase of pain threshold up to the level of surgical anesthesia providing proper instructions and suggestions to the patient ( Facco *et al.*, 2011; Facco *et al.*, 2013; Kendrick *et al.*, 2016); this is a very relevant fact allowing for enhanced recovery after surgery without adverse events ( Facco, 2016); the same is for meditation, a valuable introspective technique sharing several features with hypnosis ( Facco, 2017). However, even with these techniques, information about pain intensity and its qualities can be obtained only by 1PAs.

## Conclusions

The main aim of our paper is not that of supporting the view that the study of the biological correlates of many 1PAs is irrelevant and a waste of resources, but that the information we can gather from 1PAs are irreducible to 3PAs and these ones can only complement the information we got from 1PAs even when is it possible to infer a direct causal relationship between 3PAs and 1PAs. We recommend to read the debate with the reviewers for a more comprehensive evaluation of this claim.

Our approach is akin Jack’s (2013) statements “.. *our experiential understanding of our own minds is fundamentally different from, and at least to some degree incompatible with, our understanding of the mind as a mechanism. At the same time, this experiential understanding is no less important than our mechanistic understanding of the mind. In fact, it is more important. Our experiential perspective guides our understanding of ourselves, and serves as the compass which aids our navigation through the social world, allowing us to see, and ultimately connect to, the humanity in others*. page 670”.

Similar position is held by Guta, (2015): “.. *the knowledge [neuronal, chemical, electrical activities that take place in the brain] we gather in this regard, no matter how detailed it may turn out to be, offers no help whatsoever in and of itself by way of giving us access to the first-person data. To retrieve the latter data, the right thing to do would be to directly engage with subjects of experience, that is, with people. The imaging techniques scan brains but not people’s thoughts/intentions/plans/regrets, and the list goes on and on*. page 241”

According to the authors of “Neuromania: on the limits of brain science” ( Legrenzi & Umiltà, 2011) the popularity of the prefix “neuro” before economy ( Camerer *et al.*, 2005), aesthetics ( Skov & Vartanian, 2009), marketing ( Ariely & Berns, 2010), theology ( Barrett, 2011), etc., represents a degeneration of an acritical adhesion of a metaphysical physicalism or mind-brain identity theory and of a superficial knowledge of the complex relationship between mind contents and its neural correlates. Many authors continue to alert researchers about the problems in defining such relationship. Max Coltheart for example, repeatedly warned that “testing theories of cognition” by using fMRI investigations requires “ *both sensitivity (a claim that brain region X will always be active when cognitive process C is being executed) and specificity (the claim that brain region X will not be active except when cognitive process C is being executed). pag.102* ( Coltheart, 2013) avoiding the so-called “consistency fallacy” that is the erroneous inference that when data that are consistent with some theory they cannot, just in virtue of this consistency, be offered as the only evidence in support of that theory. Something additional is needed, that is, evidence against the contradictory of the hypothesis.

We hope this essay will alert all scientists who are endorsing a metaphysical physicalism approach who posit that all mind contents are nothing but a byproduct of the brain or emerging properties of its computational complexity ( Schwartz *et al.*, 2016; Smart, 2014) that for many phenomena, the 1PAs are the only reliable source of information available and that the knowledge of their neural and psychophysical correlates does not offer any additional information about their contents, but only complementary information. Furthermore, the wealth of data available on hypnosis and meditation see ( Facco, 2014; Facco, 2017), as well as music perception and performance ( Fauvel *et al.*, 2014; Han *et al.*, 2009; Koelsch *et al.*, 2005; Ohnishi *et al.*, 2001) provide an increasing evidence that the mind-brain relationship is not an unidirectional one, defined by a bottom-up hierarchy from brain to mind; rather, it can be better conceived as a bidirectional relationship, where mind may also engender both functional and steady, structural changes in the brain. Needless to say, music, its value and meaning, can only exist in the realm of 1PA. The whole problem is endowed with huge epistemological and metaphysical implications, to be reappraised in order to avoid any inadvertent dogmatic drift in the scientific approach to the world of subjectivity ( Klein, 2013; Klein, 2015c)

Given the enormous investments in the brain research both in the USA and Europe (see Global Brain Workshop, 2016; Markram, 2012), there is a serious risk that very few research resources (e.g. funds, personnel, etc.) will be devoted to the investigation of 1PAs. It is curious that a similar worry is shared by supporters of a mind-brain physicalism like Schwartz *et al.*, (2016), when they declare that “.. *an eliminative reductionist perspective, in which behaviours, thoughts, feelings, and other experiences can be completely explained by biological processes at the cellular and molecular levels, may be difficult to square with much current scholarship in neuroscience and in the broader field of psychology. Nevertheless, given the dependence of researchers, departments, and universities on federal grant funding, priorities emphasized by funding agencies and by their review committees may “force the hands” of researchers, departments, and universities to prioritize neuroscience at the expense of other approaches*”. Page 15

Following Stanley Klein discussion about the limitations of reducing the study of Psychological Science to its biological mechanisms, we endorse his claim that “ *experiential aspects of reality (reflected in mental construct terms such as memory, belief, thought, and desire) give us reason to remain open to the need for psychological explanation in the treatment of mind*.” ( Klein, 2016; page 357)
