# Supplementary material for: On the primacy and irreducible nature of first-person versus third-person information
Source: F1000Res. 2017 May 31;6:99. Originally published 2017 Feb 1. [Version 3] doi: 10.12688/f1000research.10752.3 (PMC5750720; doi:10.12688/f1000research.10752.3)
Supplement: Peer review history

# Peer review: Referee response for version 3

**DOI:** 10.5256/f1000research.12704.r28952

**Published:** 2018-01-02

**Authors:** Samah Khaled Zahran

**Affiliations:** 1 Personality and Social Psychologist, Ain Shams University, Cairo, Egypt

If this article is just a speculative one, it doesn't matter. But, if it is a scientific article, I think the paper needs modifications, in light of the suggested items below:

  

- What is the importance of this paper? Aim is clear, but any scientific work must add to science; to develop science. So that, I suggest if the researchers specify well the role of first and third person in perception's fields, topics. Give examples in specific research fields, and the effect of their perception types in developing science, and then relate this with previous studies and theories, in the light of definite methodology.
- In the advancement of science, all roles are important; the demand is to specify each one role, (1 ^st^ third person perceptions), in the light of related scientific works.
- Finally, scientific article depends on: Introduction, methodology, results, and conclusion after analysis.

- We suggest, reviewing this site: humancond.org/_media/papers/johannson05_third_person_effect.pdf , it's a pdf file about

 Neuwirth and Fredrick work in. (2002) who found both first- and third-person effects when studying the perceived influence of news  (example field here is in Political Science).

 - Another one: in Wikipedia, discussing the scientific topic and its history, with many references.

 - Finally, another example in the media field, at:

 http://www.rcommunicationr.org/index.php/vol-5-2017/155-mcleod-et-al-2017-thinking-about-the-media , discussing:

 Third-person Perceptions when individuals see others as being more affected by harmful media content than they are themselves, in the light of psychology, sociology and political science.

 I hope you find these helpful, with all my appreciation.

I have read this submission. I believe that I have an appropriate level of expertise to confirm that it is of an acceptable scientific standard, however I have significant reservations, as outlined above.

# Peer review: Referee response for version 3

**DOI:** 10.5256/f1000research.12704.r23093

**Published:** 2017-06-06

**Authors:** Zoltan Kekecs

**Affiliations:** 1 Lund University, Lund, Sweden

I would like to thank the authors for their detailed response and their perseverance in this debate.

 I am perfectly fine with the claims that *“at the current level of scientific advancement some first-person accounts cannot be reduced to their third-person neural and psychophysiological correlates” *and *“whichever information 3PAs can obtain related to 1PAs, this information is qualitatively different from that one experienced subjectively”.* The authors are basically saying that there is a special quality to qualia that cannot be captured by discovering its neurobiological correlates. These claims may be true and are fine in an opinion-article. Thus, it is also fine to say that *“3PAs derived from psychophysiological monitoring or neuroimaging, cannot in any way provide a more accurate idea about the qualia of the person, than the person himself experienced”*. However, this is not really the central point of the paper.

 The meat of the paper, contained in the “When first-person accounts are the only valid information to consider” section argues for something else: the superiority of **self-report of qualia **(and not experience or introspection!) vs. 3PAs on qualia. Most of the content within the paper argues for that **self-report **of qualia is the only information to reckon when it is necessary to analyse **qualia contents**.

 For example, sentences like this: *“the knowledge [neuronal, chemical, electrical activities that take place in the brain] we gather in this regard, no matter how detailed it may turn out to be, offers no help whatsoever in and of itself by way of giving us access to the first-person data. To retrieve the latter data, the right thing to do would be to directly engage with subjects of experience, that is, with people.” *directly imply that we can get access to qualia content through self-reports, but not through 3PA reports.

 To support this claim, the authors bring many examples of **self-reports** of qualia being routinely used in research and therapy, such as rating pain on a VAS, or rating emotional arousal and valence on a Self-Assessment Manikin (SAM), showing that these provide easily interpretable data about qualia. Then they bring counter-examples of brain imaging results, which are very hard to interpret in terms of their information content on qualia. This implies that **self-reports **of qualia are **the only** reliable information about **qualia content**.

 However, pain VAS ratings, and emotion SAM ratings basically consist of numbers. We can make our computer programs interpreting fMRI, EEG, or EDA data output the exact same numerical answer as we would get through these self-report ratings with certain amount of accuracy. See for example:

 Huang, G., Xiao, P., Hung, Y. S., Iannetti, G. D., Zhang, Z. G., & Hu, L. (2013). A novel approach to predict subjective pain perception from single-trial laser-evoked potentials. Neuroimage, 81, 283-293 ^1^.

 Hullett, B., Chambers, N., Preuss, J., Zamudio, I., Lange, J., Pascoe, E., & Ledowski, T. (2009). Monitoring Electrical Skin ConductanceA Tool for the Assessment of Postoperative Pain in Children?. The Journal of the American Society of Anesthesiologists, 111(3), 513-517 ^2^.

 Marquand, A., Howard, M., Brammer, M., Chu, C., Coen, S., & Mourão-Miranda, J. (2010). Quantitative prediction of subjective pain intensity from whole-brain fMRI data using Gaussian processes. Neuroimage, 49(3), 2178-2189 ^3^.

 Zhou, F., Qu, X., Helander, M. G., & Jiao, J. R. (2011). Affect prediction from physiological measures via visual stimuli. International Journal of Human-Computer Studies, 69(12), 801-819 ^4^.

 Chang, C. Y., Zheng, J. Y., & Wang, C. J. (2010, July). Based on support vector regression for emotion recognition using physiological signals. In Neural Networks (IJCNN), The 2010 International Joint Conference on (pp. 1-7). IEEE ^5^.

 Bailenson, J. N., Pontikakis, E. D., Mauss, I. B., Gross, J. J., Jabon, M. E., Hutcherson, C. A., ... & John, O. (2008). Real-time classification of evoked emotions using facial feature tracking and physiological responses. International journal of human-computer studies, 66(5), 303-317 ^6^.

 If we give a researcher 50 printouts of participant VAS ratings and 50 printouts of predicted VAS ratings based on physiological data (both a sheet of white paper with a single number on it), the researcher would have no way of knowing which of the reports came from 1PAs and 3PAs. Thus, given that we will reach a high enough accuracy in prediction, we can substitute 1PAs with 3PAs, and gain the exact same information for pain VAS ratings. And our accuracy is gradually increasing, some of the prediction algorithms already reaching r = 0.75 correlation with a single monitored psychophysiological feature.

 The authors may have the opinion that we will never reach 100% accuracy in these predictions, which is fine. If this is true, self-reports will always give us a more reliable account of qualia than 3PA-derived reports. However this is only true in cases where we can rely on self-reports. My previous review gives many examples where this may not be true. In these cases we may get a more reliable account of qualia from 3PA-derived reports. So even if the hunch of the authors is true about the possible level of predictive accuracy of self-reports with 3PA-derived reports, they still need to refine the statements to more accurately indicate that self-reports of 1PAs are superior to 3PAs only in circumstances where self-reports on 1PAs are readily available and reliable.

 I believe the following statements are also problematic, because they are unclear.

*“these first-person accounts are the only information to reckon when it is necessary to analyse qualia contents”*

*“there are many varieties of 1PAs whose contents and characteristics can be known and investigated only by these accounts and cannot be integrated with information gathered by 3PAs in particular those related to their neural or psychophysiological correlates.”*

*“the knowledge of their neural and psychophysiological correlates has nothing to add to the knowledge of these phenomena.”*

 In their current context all of these sentences imply that neurobiological accounts of qualia do not hold any information about qualia content, while self-reports do. See the added brackets:

*“these first-person accounts *[self-reports] *are the only information to reckon when it is necessary to analyse qualia contents”*

*“there are many varieties of 1PAs whose contents and characteristics can be known and investigated only by these accounts* [self-reports] *and cannot be integrated with information gathered by 3PAs in particular those related to their neural or psychophysiological correlates.”*

*“the knowledge of their neural and psychophysiological correlates has nothing to add to the knowledge of these phenomena *[over that gained through self-report].”

 However, I believe that the above mentioned example about self-reported pain on a VAS vs. computer predicted pain on a VAS based on psychophysiological parameters clearly shows that the distinction between self-reports of 1PAs and 3PA-derived reports of 1PAS are not qualitatively different. So either they both fail to convey any information on qualia content, or they both hold some information, and one is more accurate than the other (in most but not all cases, self-report is more accurate).

 On the other hand it is possible that my interpretation of these sentences is wrong, and the authors are not talking about self-report here. In this case, these sentences need to be clarified.

 One issue is that the authors still seem to use “first person accounts (1PAs)” both to indicate qualia and self-report of qualia. For example in this sentence, the phrase “1PAs” is used to refer to self-report of qualia: *“For each of the selected phenomena we will present some examples of 1PAs and 3PAs to make evident the different informational value of these accounts as supportive of our main thesis.”*. However, in the definition of “1PAs” the authors clearly say that it refers to mental content: *“First-person accounts (1PAs) are phenomenological subjective mental content the person is aware of and that can be communicate[d] to others”*. It is very important to distinguish experiences from self-reports about experiences, because experiences are inherently true and reliable, while reports on these can be untrue or unreliable because of limits in our communicational or mental abilities.

 One alternative way of interpreting the above mentioned problematic statements is if the authors meant that qualia content is only accessible to introspection (a common claim in philosophy). So the sentences would read something like this:

 “knowledge gained through introspection is the only information to reckon when it is necessary to analyse qualia contents”

 “there are many varieties of 1PAs whose contents and characteristics can be known and investigated only through introspection and cannot be integrated with information gathered by 3PAs in particular those related to their neural or psychophysiological correlates.”

 “the knowledge of their neural and psychophysiological correlates has nothing to add to the knowledge of these phenomena over and above that accessible through introspection.”

 This would make the above sentences much more defensible, because it leads back to the original first claim, that personal experiences have a certain quality that cannot be reduced to 3PAs.

 However, that would kind of invalidate the “When first-person accounts are the only valid information to consider” section where the whole argument is centered around that we need to focus more on self-reports instead of neurobiological data. So that section would also need to be changed then to argue that introspection is the preferred method of research in these cases if we want to truly study qualia content(!), while self-report and 3PA derived reports are only useful to “understand, classify, judge, etc. the mental, emotional, behavioural, perceptual subjective experience of another person”. Furthermore, the introspection interpretation would also incur the usual criticism regarding introspection methods, such as that the conscious attempts of introspection distorts experiences, and that introspection is constricted by the same limitations as every other mental activity using attentional resources.

 It is also unclear, whether the authors mean self-report of qualia, or introspection of qualia, or the experience itself in this sentence, as the “only reliable source of information”:

*“1PAs *[self-report on qualia or introspection of experiences or experiences themselves?] a *re the only reliable source of information available and that the knowledge of their neural and psychophysical correlates does not offer additional information about their contents, but only complementary information.”*

 The authors say in their reply that *‘The statement “For example if one would like to investigate whether hypnosis can induce a certain visual illusion, this technique may be used to add another layer of evidence to the claim of the subject that the visual illusion was indeed experienced, which may dispel criticism that the subject only reported the presence of the illusion to please the hypnotherapist.” is absolutely correct and this research approach is also defined neurophenomenological because it entails both 1PAs and 3PAs.’* If this statement is indeed correct, then in this research we have successfully used integrated information from self-reports of qualia and 3PAs, thus falsifying the following claim for visual illusions: *“there are many varieties of 1PAs whose contents and characteristics can be known and investigated only by these accounts and cannot be integrated with information gathered by 3PAs in particular those related to their neural or psychophysiological correlates.”*

**

 The bottom line is,

 - I still think that the manuscript needs a fair amount of clarification in terms of when are the authors talking about qualia (the subjective phenomenological experience), when are they talking about self-report of qualia, and when are they talking about introspection of experiences.

 - The limitation of using self-reports (and introspection, if this is a key feature) needs to be better highlighted

 - Claims about the superiority of self-reports vs. 3PA derived information needs to be qualified with potential limitations when self-reports cannot be relied upon

 - It needs to be clarified whether self-reports of qualia and 3PAs can provide any information about “qualia content” and what is the difference between the “analysis of qualia content” vs. “understand, classify, judge” qualia.     

 I think it is possible for us to arrive at a shared opinion, once the above things are clarified.

I have read this submission. I believe that I have an appropriate level of expertise to confirm that it is of an acceptable scientific standard, however I have significant reservations, as outlined above.

## References

[1] HuangGXiaoPHungYSIannettiGDZhangZGHuL: A novel approach to predict subjective pain perception from single-trial laser-evoked potentials. *Neuroimage*.2013;81: 10.1016/j.neuroimage.2013.05.017 283-93 10.1016/j.neuroimage.2013.05.017 23684861

[2] HullettBChambersNPreussJZamudioILangeJPascoeELedowskiT: Monitoring electrical skin conductance: A tool for the assessment of postoperative pain in children?. *Acute Pain*.2009;11(3-4) : 10.1016/j.acpain.2009.10.024 10.1016/j.acpain.2009.10.024 19672172

[3] MarquandAHowardMBrammerMChuCCoenSMourão-MirandaJ: Quantitative prediction of subjective pain intensity from whole-brain fMRI data using Gaussian processes. *Neuroimage*.2010;49(3) : 10.1016/j.neuroimage.2009.10.072 2178-89 10.1016/j.neuroimage.2009.10.072 19879364

[4] ZhouFQuXHelanderMJiaoJ: Affect prediction from physiological measures via visual stimuli. *International Journal of Human-Computer Studies*.2011;69(12) : 10.1016/j.ijhcs.2011.07.005 801-819 10.1016/j.ijhcs.2011.07.005

[5] ChangCY: Based on support vector regression for emotion recognition using physiological signals. *Neural Networks, The 2010 International Joint Conference on (IJCNN)*.2010;1-7

[6] BailensonJPontikakisEMaussIGrossJJabonMHutchersonCNassCJohnO: Real-time classification of evoked emotions using facial feature tracking and physiological responses. *International Journal of Human-Computer Studies*.2008;66(5) : 10.1016/j.ijhcs.2007.10.011 303-317 10.1016/j.ijhcs.2007.10.011

# Peer review: Referee response for version 3

**DOI:** 10.5256/f1000research.12704.r23120

**Published:** 2017-06-01

**Authors:** Maurits van den Noort, Peggy Bosch

**Affiliations:** 1 Research Group of Pain and Neuroscience, Kyung Hee University, Seoul, Korea, South; 2 Brussels Institute for Applied Linguistics, Free University of Brussels, Brussels, Belgium; 3 Donders Centre for Cognition, Radboud University Nijmegen, Nijmegen, Netherlands

We have read this submission. We believe that we have an appropriate level of expertise to confirm that it is of an acceptable scientific standard.

# Peer review: Referee response for version 2

**DOI:** 10.5256/f1000research.12464.r22513

**Published:** 2017-05-22

**Authors:** Zoltan Kekecs

**Affiliations:** 1 Lund University, Lund, Sweden

I am grateful for the detailed response of the authors and I feel that the manuscript have improved due to the amendments. However, there are some issues that still need addressing:

 The authors state in their reply that the following: “even if in the paper we mainly referred to 3P neuro- and psychophysiological correlates, even the 3PA “ *She feels happy today*” cannot convey any reliable information about how the person really feels happy. This 3PA remains an independent source of information and cannot guarantee what really the person feel. It is like to state “ *Peter felt a strong pain after hammering his thumb*”, but only Peter can describe the degree and qualities of his pain.”

 I don’t agree on the claim that “only Peter can describe the degree and qualities of his pain”. I would agree that only Peter can feel his pain in its whole entirety. Maybe it is also true that Peter will give the most accurate verbal report on the qualities of his pain. In most cases with healthy adult humans who have a good mastery of speech, who are in control of their communication faculties, and who are motivated to tell the truth this is probably true. But this is not true in all cases.

 - Consider for example a small child. I have a 1.5 years old son. He often cannot express his feelings, and I am often able to give a better verbal account of what he means about his personal experiences when he says something, than he himself. When I do this, I use information from his environment, about what happened to him or what did he do before the attempted communication about the feeling, about his behaviour, etc.

 - Another scenario: Let’s say Peter had a car accident during which he lost his ability to communicate. The arriving ambulance crew may manually probe around his body to find painful areas. For example a paramedic pressing on his leg might observe a reflexive wail and body movement. He would probably conclude that the patient experienced a sharp intense pain, indicating a fracture.

 - Furthermore, consider a physician dealing with acute injuries every day of his career for 30 years. This physician may be able to give a very accurate prediction about what a particular patient will self-report about his pain just by looking at the injuries. In many cases this is actually necessary, because the patient is not a native speaker, but can understand the language better, or because the patient does not know what qualities of the pain is relevant. So the physician will ask questions like “do you feel a sharp, stabbing pain whenever you move your wrist”? And the patient may find that this is a better description of his pain than he did, or could have reported. This is another example of when it is not only Peter who can describe the degree and qualities of his pain.

 - Also, consider the situation of a patient getting general anaesthesia including muscle relaxants. If that patient wakes up during surgery due to improper medication dose, he may be unable to communicate that he is now conscious and has pain, because the muscle relaxants block all types of movement. But the anaesthesiologist may be able to detect that he is conscious or that he is in pain using physiological and psychophysiological monitoring. There are methods for doing this even today. Although, admittedly, the level of detail these methods can provide are very crude: “the patient is approaching awareness according to the BIS reading” or “perspiration, heart rate, and blood pressure changes indicate that the patient might be in pain”. But this information is still more accurate in this particular situation than the information that we can get from a self-report.

 - And of course, people are not always motivated to tell the truth or tell the truth in the level of detail that we are interested in. It is very easy to imagine situations when a person is motivated to lie or withhold information about his or her personal experiences, for example, his memories of a crime. Clever interrogative techniques may be used in adjunct with psychophysiological and behavioural monitoring to detect discrepancies about actual memories and reports of memories.

 So there are several scenarios in which 3PAs, and even 3PAs derived from psychophysiological monitoring or neuroimaging, can provide a more accurate idea about the qualia of the person, than the person himself has provided. Some of these applications are already in use now, and we are probably going see new technological advances that would enable us to get finer details more reliably.

 My point is that the claim that “at the current level of scientific advancement, there are many varieties of 1PAs whose contents and characteristics can be known and investigated only by these accounts and cannot be integrated with information gathered by 3PAs in particular those related to their neural or psychophysiological correlates” only holds true in ideal circumstances, when dealing with adult humans who have a good mastery of communication, who are in control of their communication faculties, and who are motivated to tell the truth, and if extracting self-report is practical. This is briefly hinted upon in the section “First-person accounts are not always reliable”, but the authors conclude at the end of that section that “3PAs cannot offer a better information than those obtained by the 1PAs”. I think that in all of the above mentioned examples, 3PAs provide better information on qualia than personal reports. The manuscript could benefit from revising this section and the claims that 3PAs cannot offer a better information than those obtained by the 1PAs or that they cannot be integrated to get a clearer picture with personal reports.

 I tend to agree that at our current level of technological advancement, psychophysiological monitoring and neuroimaging techniques are so crude when it comes to providing information on qualia content that it is practically extremely unreliable and thus in almost all cases, relying on 1PAs and other non-physiological 3PA techniques are overwhelmingly more useful. But the current language of the manuscript generalizes this to every situation and not just psychophysiological monitoring/neuroimaging, but all types of 3PAs. The best way to make the claims more defendable may be to restrict them to the domain of brain and psychophysiological correlates everywhere in the manuscript. It may also help if there was more emphasis on the “exceptions”, when we are not dealing with ideal healthy humans and where personal reports are untrustworthy or not practical to extract.

 In the following I will detail some specific weaknesses of the arguments in the manuscript due to them being overly generalized.

 - In medicine we are routinely using observer rating scales (3PAs) to judge subjective pain experiences of children and people living with mental disabilities preventing them from communication. Similarly, as mentioned above, physiological indices are also used routinely in surgery to judge awareness level and pain in anaesthesia. Furthermore, we are already capable of giving a quite accurate pain intensity prediction just by using fMRI data. See for example:

 Marquand, A., Howard, M., Brammer, M., Chu, C., Coen, S., & Mourão-Miranda, J. (2010). Quantitative prediction of subjective pain intensity from whole-brain fMRI data using Gaussian processes. *Neuroimage*, *49*(3), 2178-2189 ^1^

 So 3PAs and even fMRI data can provide useful information on qualia content, and yet, the authors claim that “information about pain intensity and its qualities can be obtained only by 1PAs”. This claim is too general in its current form.

 - Similar claims are made about emotions: “Emotions identification and their valence and arousal can be measured only taking in account 1PAs.“ However, automatic emotion classifying algorithms show a pretty good match with subjective reports of emotional states.

 Zhou, F., Qu, X., Helander, M. G., & Jiao, J. R. (2011). Affect prediction from physiological measures via visual stimuli. *International Journal of Human-Computer Studies*, *69*(12), 801-819 ^2^.

 Chang, C. Y., Zheng, J. Y., & Wang, C. J. (2010, July). Based on support vector regression for emotion recognition using physiological signals. In *Neural Networks (IJCNN), The 2010 International Joint Conference on* (pp. 1-7). IEEE ^3^.

 Bailenson, J. N., Pontikakis, E. D., Mauss, I. B., Gross, J. J., Jabon, M. E., Hutcherson, C. A., ... & John, O. (2008). Real-time classification of evoked emotions using facial feature tracking and physiological responses. *International journal of human-computer studies*, *66*(5), 303-317 ^4^.

 Knowledge gained about emotional states from these physiological monitoring techniques can be integrated with personal reports in research as well. Getting personal reports is often not practical or possible. For example if a researcher wants to monitor affective changes during hypnosis or under the effects of psychoactive drugs, personal reports might be unreliable or asking for self-monitoring such as this might even change the whole experience of the participant.

 - We are at a point where we can even make above chance predictions of what type of item a person is imagining using just fMRI data. See for example:

 Reddy, L., Tsuchiya, N., & Serre, T. (2010). Reading the mind's eye: decoding category information during mental imagery. *NeuroImage*, *50*(2), 818-825 ^5^.

 So it is possible to extract information about the contents and the subjective experience of a person during a visualization task, and it is easy to see how this might generalize to memory tasks. Presently, using this technique is hardly practical, but it shows that a “mind reading” of sorts is possible, and it might even become practical or even cost effective to do in some situations as technology advances. For example, this type of technology might be used to enable communication for those incapable of movement, or to probe the needs of people with mental or communication disabilities.

 - Neural mechanisms correlated with visual illusions are also well explored. See for example:

 Pan, Y., Wang, L., Wang, Z., Xu, C., Yu, W., Spillmann, L., ... & Wang, W. (2016). Representation of illusory and physical rotations in human MST: A cortical site for the pinna illusion. Human brain mapping ^6^.

 In fact, we can already predict whether a person is experiencing an illusion or not using neuroimaging techniques:

 Schwarzkopf, D. S., Song, C., & Rees, G. (2011). The surface area of human V1 predicts the subjective experience of object size. Nature neuroscience, 14(1), 28-30 ^7^.

 Andrews, T. J., Schluppeck, D., Homfray, D., Matthews, P., & Blakemore, C. (2002). Activity in the fusiform gyrus predicts conscious perception of Rubin's vase–face illusion. Neuroimage, 17(2), 890-901 ^8^.

 Chen, L. M., Friedman, R. M., & Roe, A. W. (2003). Optical imaging of a tactile illusion in area 3b of the primary somatosensory cortex. Science, 302(5646), 881-885 ^9^.

 We are also able to describe which image is a person or an animal is currently experiencing in a binocular rivalry task just from fMRI data:

 Haynes, J. D., & Rees, G. (2005). Predicting the stream of consciousness from activity in human visual cortex. *Current Biology*, *15*(14), 1301-1307 ^10^.

 Furthermore, we can directly induce or diminish illusions by stimulating the correct neural structures. See for example:

 Antal, A., Varga, E. T., Nitsche, M. A., Chadaide, Z., Paulus, W., Kovács, G., & Vidnyánszky, Z. (2004). Direct current stimulation over MT+/V5 modulates motion aftereffect in humans. Neuroreport, 15(16), 2491-2494 ^11^.

 Kammers, M. P., Verhagen, L., Dijkerman, H. C., Hogendoorn, H., De Vignemont, F., & Schutter, D. J. (2009). Is this hand for real? Attenuation of the rubber hand illusion by transcranial magnetic stimulation over the inferior parietal lobule. Journal of Cognitive Neuroscience, 21(7), 1311-1320 ^12^

 So we are getting better and better in getting information on a person’s experience content, and even inducing experiences by exploiting our knowledge of the neural correlates of subjective experiences. This type of knowledge can actually “help to increase what authors already know” about the hallucinations of their participants. For example if one would like to investigate whether hypnosis can induce a certain visual illusion, this technique may be used to add another layer of evidence to the claim of the subject that the visual illusion was indeed experienced, which may dispel criticism that the subject only reported the presence of the illusion to please the hypnotherapist. Also it might not be practical to constantly probe the experience of the participant in a binocular rivalry task, in which case the brain imaging information might serve as a good enough alternative to monitor changes in visual experiences.

 The bottom line is that in my opinion, several of the claims of the authors remain vulnerable to criticism in this version 2 of the manuscript, because the claims are still too general. The claims could be revised to imply that self-reports of 1PAs are superior to 3PAs in circumstances where self-reports on 1PAs are readily available and reliable. In cases where self-reports on 1PAs cannot be fully relied upon or are not available, 3PAs might prove a good enough surrogate. I advise a revision of the manuscript to make this point clear, or to refute this line of criticism. (This actually affects most of the claims made by the authors because most of them imply that 3PAs are always inferior to self-reports or that self-reports are the only way to study particular 1PAs.)

 The authors modified the definition of 1PAs in this revised version of the manuscript to “phenomenological subjective mental content the person is aware of and that can be communicate to others, if requested or desired, by written, verbal or intentional (conscious) behaviour”. Due to this change some of the previous claims make more sense. But other claims now are less clear, and need to be re-written with the new definition of 1PAs in mind. For example, claim a)”some 1PAs cannot be reduced to third-person neural and psychophysiological correlates accounts (3PAs)” make more sense now, because it says that some qualia are irreducible to neural events. However, claim b) “their contents are the only information to reckon when it is necessary to analyse qualia contents, that is, emotions, beliefs, reality interpretations, quality of life and health and their effects on behaviour and the brain activity” became less informative now. Now it basically means that we only need information about the content of (communicable) qualia, if we want to know the contents of qualia. This is self-evident. I guess the authors meant that contents of self-reports of qualia are the only information to reckon when it is necessary to analyse qualia contents. I suggest that the authors make sure that they clearly differentiate whenever they use the word 1PA or 1PAs, whether they actually mean qualia, or self-report of qualia.

 I still feel that the statement “the knowledge of their neural and psychophysiological correlates has nothing to add to the knowledge of these phenomena” is too general. I suggest revising it to be more specific, for example: “the knowledge of their neural and psychophysiological correlates has nothing to add to our knowledge of the content of personal experiences, when there are reliable self-reports available.” See above arguments and my version 1 comment on this as well for reasoning.

 The authors have amended the section talking about the effects of the interventions on their biological correlates of qualia. Now they say: “Saitoh et al. (2007) were successful in reducing pain due to spinal cord or peripheral lesions by applying high-frequency repetitive transcranial magnetic stimulation on the primary motor cortex. However, the modification of primary cortex activity didn’t give any useful information about the participants’ change in pain perception. In fact, this information was obtained by asking the participants to rate their pain with a visual analogue scale similar to that presented in Figure 2 and the Short-Form of the McGill Pain Questionnaire.”

 I feel that this line of argument could be strengthened. Saitoh et al. predicted the reduction in pain intensity in their subjects. That is why they did the stimulation in the first place. A targeted modification to the neural correlate of the 1PA managed to change the 1PA. To me this still seems to refute the claim that 1PAs are irreducible to neural correlates. I wonder whether it would be worthwhile to supplement this argument with the suggestion that the transcranial magnetic stimulation did not directly change the qualia itself (1PA), rather, it affected some neural precursor that eventually led to the qualia. Similarly as local anaesthesia can hardly be claimed to change the structures evoking qualia directly, likewise it is not certain, that transcranial magnetic stimulation directly affected the experience of pain, it is possible that its effect was more indirect.

I have read this submission. I believe that I have an appropriate level of expertise to confirm that it is of an acceptable scientific standard, however I have significant reservations, as outlined above.

## References

[1] MarquandAHowardMBrammerMChuCCoenSMourão-MirandaJ: Quantitative prediction of subjective pain intensity from whole-brain fMRI data using Gaussian processes. *Neuroimage*.2010;49(3) : 10.1016/j.neuroimage.2009.10.072 2178-89 10.1016/j.neuroimage.2009.10.072 19879364

[2] ZhouFQuXHelanderMJiaoJ: Affect prediction from physiological measures via visual stimuli. *International Journal of Human-Computer Studies*.2011;69(12) : 10.1016/j.ijhcs.2011.07.005 801-819 10.1016/j.ijhcs.2011.07.005

[3] ChangCYZhengJYWangCJ: Based on support vector regression for emotion recognition using physiological signals. *In Neural Networks (IJCNN), The 2010 International Joint Conference IEEE*.2010;1-7

[4] BailensonJPontikakisEMaussIGrossJJabonMHutchersonCNassCJohnO: Real-time classification of evoked emotions using facial feature tracking and physiological responses. *International Journal of Human-Computer Studies*.2008;66(5) : 10.1016/j.ijhcs.2007.10.011 303-317 10.1016/j.ijhcs.2007.10.011

[5] ReddyLTsuchiyaNSerreT: Reading the mind's eye: decoding category information during mental imagery. *Neuroimage*.2010;50(2) : 10.1016/j.neuroimage.2009.11.084 818-25 10.1016/j.neuroimage.2009.11.084 20004247PMC2823980

[6] PanYWangLWangZXuCYuWSpillmannLGuYWangZWangW: Representation of illusory and physical rotations in human MST: A cortical site for the pinna illusion. *Hum Brain Mapp*.2016;37(6) : 10.1002/hbm.23156 2097-113 10.1002/hbm.23156 26945511PMC6867246

[7] SchwarzkopfDSSongCReesG: The surface area of human V1 predicts the subjective experience of object size. *Nat Neurosci*.2011;14(1) : 10.1038/nn.2706 28-30 10.1038/nn.2706 21131954PMC3012031

[8] AndrewsTSchluppeckDHomfrayDMatthewsPBlakemoreC: Activity in the Fusiform Gyrus Predicts Conscious Perception of Rubin's Vase–Face Illusion. *NeuroImage*.2002;17(2) : 10.1006/nimg.2002.1243 890-901 10.1006/nimg.2002.1243 12377163

[9] ChenLMFriedmanRMRoeAW: Optical imaging of a tactile illusion in area 3b of the primary somatosensory cortex. *Science*.2003;302(5646) : 10.1126/science.1087846 881-5 10.1126/science.1087846 14500850

[10] HaynesJDReesG: Predicting the stream of consciousness from activity in human visual cortex. *Curr Biol*.2005;15(14) : 10.1016/j.cub.2005.06.026 1301-7 10.1016/j.cub.2005.06.026 16051174

[11] AntalAVargaENitscheMChadaideZPaulusWKovácsGVidnyánszkyZ: Direct current stimulation over MT+/V5 modulates motion aftereffect in humans. *NeuroReport*.2004;15(16) : 10.1097/00001756-200411150-00012 2491-2494 10.1097/00001756-200411150-00012 15538181

[12] KammersMPVerhagenLDijkermanHCHogendoornHDe VignemontFSchutterDJ: Is this hand for real? Attenuation of the rubber hand illusion by transcranial magnetic stimulation over the inferior parietal lobule. *J Cogn Neurosci*.2009;21(7) : 10.1162/jocn.2009.21095 1311-20 10.1162/jocn.2009.21095 18752397

# Response

**Published:** 2017-05-24

**Authors:** Patrizio Tressoldi

**Affiliations:** Dipartimento di Psicologia Generale, Università di Padova, Italy

We appreciate the efforts to rebut our strong and basic claim that 1PAs, that is to say qualia and subjective phenomenological mental and emotional experiences, cannot be reduced to 3PAs.

However, all examples given to falsify this basic claim, are based on a misunderstanding related to the difference between 1PAs contents and 1PAs interpretation, classification, etc., by using both observational and physiological 3PAs.

The examples like  “ *He (the 1.5 years old son) often cannot express his feelings, and I am often able to give a better verbal account of what he means about his personal experiences when he says something, than he himself. When I do this, I use information from his environment, about what happened to him or what did he do before the attempted communication about the feeling, about his behaviour, etc*.”

or “ *For example a paramedic pressing on his leg might observe a reflexive wail and body movement. He would probably conclude that the patient experienced a sharp intense pain, indicating a fracture*.” and “ *consider the situation of a patient getting general anaesthesia including muscle relaxants. If that patient wakes up during surgery due to improper medication dose, he may be unable to communicate that he is now conscious and has pain, because the muscle relaxants block all types of movement. But the anaesthesiologist may be able to detect that he is conscious or that he is in pain using physiological and psychophysiological monitoring*.”

And the references related on how in medicine it is possible “ *to judge subjective pain experiences of children and people living with mental disabilities preventing them from communication” *and “ *to judge awareness level and pain in anaesthesia*” etc., are all examples where a third person tries to understand, classify, judge, etc. the mental, emotional, behavioural, perceptual subjective experience of another person. Does the information obtained by 3PAs convey precisely what the kid, the patient, etc. is experiencing or did he experience?

In theory, this could be obtained by some people with high levels of empathy, but for sure not from

*“ 3PAs derived from psychophysiological monitoring or neuroimaging, *[that can, added *] provide a more accurate idea about the qualia of the person, than the person himself has provided”.*

This reviewer’s opinion is exactly the opposite of that one we are proposing in the paper, namely that 3PAs derived from psychophysiological monitoring or neuroimaging, **cannot in any way** provide a more accurate idea about the qualia of the person, than the person himself experienced.

The statement “ *For example if one would like to investigate whether hypnosis can induce a certain visual illusion, this technique may be used to add another layer of evidence to the claim of the subject that the visual illusion was indeed experienced, which may dispel criticism that the subject only reported the presence of the illusion to please the hypnotherapist.” *is absolutely correct and this research approach is also defined neurophenomenological because it entails both 1PAs and 3PAs.

Nowhere in the paper we claimed that 1PAs cannot be observed, interpreted, classified, etc. by using 3PAs. Our basic claim is that whichever information 3PAs can obtain related to 1PAs, this information is **qualitatively different from that one experienced subjectively.**

In the new version of the paper we added Table 1 in the Discussion to help to understand better the different nature and hence the irreducibility of 1PAs with respect to 3PAs.

Furthermore, in the discussion we revised the statement “the 1PAs are the only reliable source of information available and that the knowledge of their neural and psychophysical correlates **does not offer any additional information about their contents, but only complementary information.**

At this point of the (stimulating) debate we think that it is difficult for us to get a shared opinion, but we could agree the materials (paper plus our debate) at disposal of the readers (in the discussion we added “We recommend to read the debate with the reviewers for a more comprehensive evaluation of this statement”) are sufficient to let them examine the strengths and weaknesses of our statements so as to form an independent opinion.

# Peer review: Referee response for version 2

**DOI:** 10.5256/f1000research.12464.r22512

**Published:** 2017-05-11

**Authors:** Maurits van den Noort, Peggy Bosch

**Affiliations:** 1 Research Group of Pain and Neuroscience, Kyung Hee University, Seoul, Korea, South; 2 Brussels Institute for Applied Linguistics, Free University of Brussels, Brussels, Belgium; 3 Donders Centre for Cognition, Radboud University Nijmegen, Nijmegen, Netherlands

The authors did a good job and we have no further comments.

We have read this submission. We believe that we have an appropriate level of expertise to confirm that it is of an acceptable scientific standard.

# Peer review: Referee response for version 1

**DOI:** 10.5256/f1000research.11593.r21505

**Published:** 2017-04-28

**Authors:** Zoltan Kekecs

**Affiliations:** 1 Lund University, Lund, Sweden

The authors present an opinion article summarizing information from prior literature in defence of their claims that a) some first-person accounts cannot be reduced to their third-person neural and psychophysiological correlates and b) that these first-person accounts are the only information to reckon when it is necessary to analyse qualia contents. I believe this is an important topic to discuss, even if I am sceptical about whether the issue underlying the arguments, the reducibility of qualia to psychophysiological information, is one that can be decided just through scientific insight. Nevertheless, I feel that the manuscript needs a thorough revision before being finalized, making the claims themselves and the logic of the arguments supporting them clearer.

 The authors make several claims in this opinion article. One of the main points is summarized well in the Abstract and Discussion: "for many phenomena, first-person accounts are the only reliable source of information available and the knowledge of their neural and psychophysical correlates don’t offer any additional information about them”. Based on the information presented by the authors, I tend to agree with this statement if we start the sentence with “at this point in time” or “at our current level of scientific advancement”.

 It is true that presently our neuroimaging and electrophysiological monitoring techniques used in humans are extremely crude and come nowhere close to providing the level of detail that can be gained from a first person account on most of the listed phenomenon. However, the author’s claim doesn’t seem to stop at the present time. They seem to argue that third person accounts will never provide reliable information about the listed phenomena. This is problematic because this part of their claim is not justified by any arguments. Instead, the authors seem to extrapolate from the fact that third person accounts are unreliable today to the claim that they will always be unreliable and redundant compared to first person accounts. I think that this logical jump is too much to ask of the reader. Thus, either the claim should be restricted in time, or further argumentation is necessary.

 Another issue with the manuscript in its current form is that 1PAs and subjective experiences (qualia), are often confused. For example in this sentence: “Our statement that 1PAs are irreducible to 3PAs…”. I like that the authors take the time to define both first person accounts and third person account in the beginning of the paper. However, the claim that 1PAs are irreducible to 3PAs are refuted by the very definition that the authors provide. 1PA is defined as: “First-person accounts (1PAs) are written, verbal or intentional (conscious) behaviour, e.g. sign language, accounts related to what a person feels, perceives or thinks, in other words, every mental content the person is aware of and can communicate to others if requested or desired.” While 3PA is defined as: “third-person accounts (3PAs), are identical types of accounts plus their neuro and psychophysiological correlates, obtained by people who observe or measure other behaviour and mental contents and processes.“ An example for a 1PA by the authors is if a person says “I feel happy today”. This accounts can always be directly transformed to a 3PA like: “She feels happy today”. So in this sense a 1PA can be “reduced” or made directly equivalent to a 3PA.

 At another point in the manuscript the authors use a longer version of this claim: “some 1PAs cannot be reduced to third-person neural and psychophysiological correlates accounts”. However, this cannot be true either with the current definition the authors have for 1PA, because all of the examples the authors bring for 1PA can be reduced to muscle movement (speech, writing, sign language), and it is well established that muscle movements are directly evoked by neurobiological phenomenon. So it is logically possible to completely reduce the movements produced when a person utters “I have a throbbing pain in my temple” verbally or in sign language to its efferent neural source. In fact, we understand the processes that are at play here so well that we can create an artificial limb with which an arm amputee will become able to produce the same sign language sentence on her own again. So I would venture that reducing these reports themselves to their neurobiological correlates is not only a logical possibility, but is plausible within a few years of research.

 I guess what the authors really meant is that the qualia, the subjective feeling of happiness or pain, the feeling that the 1PA refers to, can never be reduced to simple 3PAs (and because of the above argument about the equivalence of 1PAs and 3PAs, it cannot be reduced to 1PAs either if we define 1PAs as the authors do right now). So either the definition of 1PAs needs to be changed to involve the subjective feeling and not just the report of that feeling, or the manuscript needs to be looked over carefully to identify sections where the authors meant qualia (first person experiences) instead of first person reports about qualia.

 I also feel that several statements and claims in the manuscript could be clarified. For example, the authors claim that “the knowledge of their neural and psychophysiological correlates has nothing to add to the knowledge of these phenomena”. This statement is very general in its current form and the preceding text does not justify it. Let’s take for example pain or mood disorders, example phenomena brought up by the authors. I believe we have gained extremely useful knowledge already about these phenomenon by understanding the neural and biochemical mechanisms involved in them, which help us in their respective treatment. We are able to further improve our treatments by understanding the mechanisms even better. I am sure that the authors did not mean that we cannot learn anything useful about these phenomenon by studying their neural correlates. They probably meant that we do not get any useful information on the exact quality of the subjective experiences involved in these phenomenon by studying their neuronal correlates, or something similar. If so, the original sentence needs much clarification.

 The quote from Coltheart (2013) is also misleading: ‘“testing theories of cognition” by using fMRI investigations requires “both sensitivity (a claim that brain region X will always be active when cognitive process C is being executed) and specificity (the claim that brain region X will not be active except when cognitive process C is being executed).”’ I don’t think any brain researcher today would think that a certain area of the brain would be responsible for a single thought or idea and nothing else. This is not even true for individual neurons. It is the networks and connections that are proposed to do the computations, and a brain area and even individual neurons are suspected to be part of multiple networks. So in this sense we cannot and do not expect this kind of specificity of brain areas anymore.

 It is strange that the authors bring up a fact that falsifies one of their claims and then they never explain why this falsification is invalid. It is left hanging in the air: “Our statement that 1PAs are irreducible to 3PAs, could be falsified by the evidence suggesting that it is possible to change 1PAs by acting on their biological correlates. For example Saitoh et al. (2007) were successful in reducing pain due to spinal cord or peripheral lesions by applying high-frequency repetitive transcranial magnetic stimulation on the primary motor cortex.” Later they add: “As a result, the 1PA is no less relevant than 3PA, even in the context of the pragmatic approach of clinical medicine, despite having been understated by the ruling reductionist paradigm.” However, this is nowhere near as strong a claim as the original one. The original claim is that 1PAs are irreducible to 3PAs, while the later claim is that 1PAs are relevant as well, not just 3PAs. By leaving the falsification open like this the authors practically invalidate one of their main claims in this opinion article, so it is strange why they make this claim the first place, if they think it is in fact false, or incomplete in its original form. I suggest either refuting the falsification, or elaborating their claim in its original form (at every instance of its appearance in the manuscript, not just after the falsification is mentioned in the end), so it is no longer falsified by the fact that it is possible to change 1PAs by acting on their biological correlates.

 Relatedly, I found it unclear how does the fact that hypnosis brings about an increase in pain threshold relate to the same section. For me, this sentence in its context without any further explanation implied that the authors think that contrary to transcranial magnetic stimulation, hypnosis would affect 1PAs directly, without involving neural correlates of pain. This is not true, because we see from several neuroimaging studies that the brain behaves differently when noxious stimuli is applied with and without hypnosis. It is also very probable that hypnosis relies on at least some neural mechanisms to enact its effects on pain, if nothing else, by relying on the sensory neurons which allow the hypnosis participant to perceive the words of the hypnotherapist. If this sentence is important in the manuscript, the authors should make it clear how it is relevant exactly to this discussion. Otherwise I suggest deleting it because it invites misinterpretation.  

 It is also hard to see how does the following sentence help any of the arguments of the authors “In fact in the Saitoh et al. (2007) example, the modification of primary cortex activity do not contain any useful information about the participants’ change in pain perception.” Transcranial magnetic stimulation was an experimental manipulation in this example. I am not sure why should it contain any information on the change in pain perception. This is not a measurement, but a manipulation targeting the suspected mechanism underlying pain, which in the end was successfully able to modify the subjective pain experience, or at least the 1PA thereof. Similarly, a hammer blow does not have to contain information on the subjective quality of pain to cause pain. If the authors meant this sentence to refute the falsification, they need to make it clearer.

 Correcting the following minor issues should also serve to improve the manuscript:

 “opioids are only wake hallucinogens” – weak instead of wake

 “according to classical, Newtonian physics” – according instead of acording

 “content that can only measured by using 1PAs” – content that can only be measured by using 1PAs

 Irreducible is spelled incorrectly as irriducible several time in the document

 “this is a very relevant fact allowing for Enhanced Recovery After Surgery without costs” – why is Enhanced Recovery After Surgery capitalized? Furthermore, “without costs” indicates that this is a completely free intervention, however, most surgeries do not have a trained professional who can use hypnosis in a clinical setting, so in most cases this would require the presence of a new professional, who needs to be paid. And even if the medical staff gets the proper training, the training itself is not without costs, etc. So I suggest deleting “without costs” from this sentence.

I have read this submission. I believe that I have an appropriate level of expertise to confirm that it is of an acceptable scientific standard, however I have significant reservations, as outlined above.

# Response

**Published:** 2017-04-30

**Authors:** Patrizio Tressoldi

**Affiliations:** Dipartimento di Psicologia Generale, Università di Padova, Italy

Thank you for your accurate and constructive review.

In the following, we will try to reply to all your main comments.

- "for many phenomena, first-person accounts are the only reliable source of information available and the knowledge of their neural and psychophysical correlates don’t offer any additional information about them”. Based on the information presented by the authors, I tend to agree with this statement if we start the sentence with “at this point in time” or “at our current level of scientific advancement”.

 

**Reply: we added the sentence “at the current level of scientific advancement” both in the abstract and in the discussion.**

 

- Another issue with the manuscript in its current form is that 1PAs and subjective experiences (qualia), are often confused…..An example for a 1PA by the authors is if a person says “I feel happy today”. This accounts can always be directly transformed to a 3PA like: “She feels happy today”. So in this sense a 1PA can be “reduced” or made directly equivalent to a 3PA.

 

**Reply: even if in the paper we mainly referred to 3P neuro- and psychophysiological correlates, even the 3PA “ *She feels happy today*” cannot convey any reliable information about how the person really feels happy. This 3PA remains an independent source of information and cannot guarantee what really the person feel. It is like to state “ *Peter felt a strong pain after hammering his thumb*”, but only Peter can describe the degree and qualities of his pain**.

 

- …So either the definition of 1PAs needs to be changed to involve the subjective feeling and not just the report of that feeling,

**Reply: in the Introduction we defined better our 1PA definition adding the term “qualia” with a reference.**

 

- I am sure that the authors did not mean that we cannot learn anything useful about these phenomenon by studying their neural correlates. They probably meant that we do not get any useful information on the exact quality of the subjective experiences involved in these phenomenon by studying their neuronal correlates, or something similar.

 

**Reply: This is precisely our core message. In fact, in the discussion we wrote “The main aim of our paper is not that of supporting the view that the study of the biological correlates of many 1PAs is irrelevant and a waste of resources, but that the information we can gather from 1PAs are irreducible to 3PAs and these ones cannot increase the information we got from 1PAs even when is it possible to infer a direct causal relationship between 3PAs and 1PAs.”**

 

- The quote from Coltheart (2013) is also misleading:…. ’ I don’t think any brain researcher today would think that a certain area of the brain would be responsible for a single thought or idea and nothing else.

 

**Reply: Coltheart’s et al. warnings are valid for any correlation between cognitive functions and their anatomical correlates independently if these ones are single or a network of brain areas. Unfortunately, these warnings are still largely ignored see for example.Tressoldi, P. E., Sella, F., Coltheart, M., & Umilta, C. (2012). Using functional neuroimaging to test theories of cognition: A selective survey of studies from 2007 to 2011 as a contribution to the Decade of the Mind Initiative. Cortex, 48(9), 1247-1250.**

 

- It is strange that the authors bring up a fact that falsifies one of their claims and then they never explain why this falsification is invalid ……… I suggest either refuting the falsification, or elaborating their claim in its original form (at every instance of its appearance in the manuscript, not just after the falsification is mentioned in the end), so it is no longer falsified by the fact that it is possible to change 1PAs by acting on their biological correlates.

**Reply: we agree that the suggestion on how to falsify our main claim and the Saitoh example was badly presented. Now we revised that paragraph as follow: “Our statement that 1PAs are irreducible to 3PAs, could be falsified by the evidence that it is possible to determine precisely the changes and qualities of 1PAs only by observing the effects of the interventions on their biological correlates. For example, Saitoh et al. (2007) were successful in reducing pain due to spinal cord or peripheral lesions by applying high-frequency repetitive transcranial magnetic stimulation on the primary motor cortex. However, the modification of primary cortex activity didn’t give any useful information about the participants’ change in pain perception. In fact, this information was obtained by asking the participants to rate their pain with a visual analogue scale similar to that presented in Figure 2 and the Short-Form of the McGill Pain Questionnaire.**

 

- .. the authors think that contrary to transcranial magnetic stimulation, hypnosis would affect 1PAs directly, without involving neural correlates of pain. This is not true,….

 

**Reply: the example of hypnosis as a mean to influence pain perception has now be integrated with the Saitoh example: “Pain reduction can also be obtained by acting on mental beliefs and contents…..**

# Peer review: Referee response for version 1

**DOI:** 10.5256/f1000research.11593.r21807

**Published:** 2017-04-11

**Authors:** Maurits van den Noort, Peggy Bosch

**Affiliations:** 1 Research Group of Pain and Neuroscience, Kyung Hee University, Seoul, Korea, South; 2 Brussels Institute for Applied Linguistics, Free University of Brussels, Brussels, Belgium; 3 Donders Centre for Cognition, Radboud University Nijmegen, Nijmegen, Netherlands

In the present opinion article ^1^, the authors firstly present support for the claim that some first-person accounts (1PAs) cannot be reduced to third-person neural- and psychophysiological correlates accounts (3PAs). Secondly, they state that the 1PAs contents are the only information to reckon when it is necessary to analyze qualia contents (e.g., emotions, beliefs, reality interpretations, quality of life and health) and their effects on behavior and the brain activity. Thirdly, according to the authors, even a complete description of the brain and psychophysiological correlates of these 1PAs does not add any further information about their contents and characteristics. Tressoldi *et al.* (2017) makes several challenging and highly interesting claims; moreover, they give a state of the art overview of the 1PAs and 3PAs results and research limitations so far. Last but not least, their article stimulates further discussion on how to best invest research money in order to make progress in this research field, all in all, we recommend publication, but we have several major and minor points that the authors should further address.

 Firstly, the authors are right (see page 2) that 1PAs are useful in clinical research and diagnostics of psychiatric disorders because they provide subjective and qualitative information; however, on the other hand, we would like to stress that self-rating instruments, such as the Beck Depression Inventory-II (BDI-II) ^2^, have their own limitations ^3^. For instance, the interpretation of results from self-report instruments in general but also for specific questionnaires, can contain flaws (e.g., Subjective Well-being under Neuroleptics scale – Short form, etc.) ^4^. For instance, it was shown that patients might show a certain response pattern, like a tendency to exaggerate their symptoms or on the contrary, to willingly under-report the severity of their symptoms or the frequency in order to present their situation more positively ^3^. In addition, test-taking attitude (e.g., social desirability) was found to play a critical role in the responses to clinical self-report instrument ^5^. In other words, how should psychologists/psychiatrists deal with those methodological limitations in daily clinical practice according to the authors? In our opinion, relying on those (structured) questionnaire outcomes only, does not seem the way to go.

 The authors are right when they write on page 3 about 3PAs: “It seems clear that this type of information cannot convey any useful information about the subjective quality of pain of the persons experiencing it”. However, the authors somehow do not mention that in the years after the Pariente *et al*. (2005) ^6^ publication, the measurement of *deqi* scores of the participants in acupuncture studies were introduced and are now being collected alongside the 3PAs ^7^^-^^8^, consisting of the following 12 *Deqi* sensations: aching, soreness, numbness, fullness, sharp or dull pain, pressure, heaviness, warmth, coolness, tingling, itching, and any others ^7^^-^^8^. This methodology is also used in recent functional magnetic resonance imaging (fMRI) studies on acupuncture ^9^; moreover, the MR signals of the brain areas that had been activated by acupuncture stimulation at a specific acupuncture point (for instance GB34) are then correlated ^9^. The authors should add this to their manuscript because this would give a more complete picture of the current state of the art in this specific research field, especially since they attack this field for using 3PAs only, which is not correct.

 Thirdly, one of their most provocative statements is the one on page 5 where the authors state: “However it is important to realize, on the part of both researchers and the funders of their investigations, that the knowledge of their neural and psychophysiological correlates *has nothing to add* to the knowledge of these phenomena”. We find this a challenging statement and we fully agree with the authors that both funding agencies and researchers are often not critical enough in their assessments of those studies and large grant applications. To date, the studies on neural and psychophysiological correlates not at all contribute significantly, taking into account the large amounts of research funding/resources that have been invested so far. However, why do the authors think that it would be technically, hypothetically, impossible to combine 1PAs with 3 PAs? Perhaps neural and psychophysiological measurements while the person is aware of and can communicate the mental contents to others (if requested or desired) could still add important clinical information (e.g., neural and psychophysiological measurements while patients with depression fill in the BDI-II)? In line with this, the authors write on page 5 in response to the Urry *et al*. (2004) ^10^ study “May we use this information to gather more details about what already participants reported in their 1PAs?”, but despite mentioning this question, they further ignore this. In our opinion, they too easily dismiss this option. Therefore, in our opinion, their statement that the neural and psychophysiological correlates “have nothing to add” to the knowledge of these phenomena is too strict and too premature, it might be right, but it could also be totally wrong.

 The fourth major point that we would like to tap (see page 6) is the fact that in their discussion the authors focus on the usefulness of biological correlates of 1PAs only. It is true that the biological perspective (significantly marked by the advances in neuroimaging techniques ^11^) is very popular in psychology at the moment; however, we are wondering what the opinion of the authors is with respect to their claims, in terms of the fundamental laws of physics ^12^? Note that to date, a unified brain processing theory (unifying physics and neuroscience) does not exist[ref-13)? How do the authors think that a better theory of its underlying fundamental laws of physics could describe and explain 1PAs and 3PAs? This area might build a bridge in the understanding of 1PAs and the underlying mechanisms that are partly measured by 3PAs.

 Finally, there are several minor issues that we would like for the authors to address in their final version of the paper. For instance, the authors should add suitable references behind “Beck Depression Inventory” and “The Structured Clinical Interview for DSM-5 (SCID-5)” (see page 2); moreover, the authors should include higher resolution images of Figure 1 and Figure 2 (see page 3). The authors should write out “NDEs” the first time that they use this abbreviation (see page 4). The easiest way seems to include “NDE” immediately after “Near-Death-Experiences” on page 4. In addition, the authors should take a closer look at “Klein (2015) ^14^” on page 4 because there are 3 “Klein (2015)” references (Klein, 2015a ^14^; Klein, 2015b ^15^; Klein, 2015c[ref16]) but the authors only use “2015a” and “2015b” (see also the reference list on page 8). Furthermore, the authors should include suitable references behind “space-time and matter-energy”, “Heisenberg’s principle of indetermination” (note it should be “Heisenberg’s” instead of “Heisnberg’s”), and “the concept of entanglement” in order to support their statements (see page 4). The authors should correct the following misspellings/errors on page 2: “be” should be added to the sentence “We will not enter here in the debate about how 1 PAs can also be considered 3PAs”, on page 4: “acording” should be replaced by “according” (see the Reasoning subsection), and on page 5: “helped” should be replaced by “help” and “knew” should be replaced by “know” (see the Hallucinations subsection), “be” should be added to the sentence “that can only be measured” (see the Risk perception subsection), and it should be “which” instead of “witch” (see the Discussion section). Also we would suggest adding a “Conclusion section” to the paper at the end of their paper (on page 6) and or alternatively at the end of the Introduction section of their paper. The last minor revision is that the authors should add a “s” behind the word “author” in the Author contributions section of their paper.

 To conclude, the present opinion article ^1^ is definitely worth publishing and will stimulate further discussion on how to best investigate and use research money and resources in the study of 1PAs and 3PAs. Moreover, the future will show whether the authors are correct in their claim that even a complete description of the brain and psychophysiological correlates of these 1PAs does not add any further information about their contents and characteristics.

We have read this submission. We believe that we have an appropriate level of expertise to confirm that it is of an acceptable scientific standard, however we have significant reservations, as outlined above.

## References

[1] TressoldiPFaccoELucangeliD: On the primacy and irreducible nature of first-person versus third-person information. *F1000Research*.2017;6: 10.12688/f1000research.10752.1 10.12688/f1000research.10752.1 29333233PMC5750720

[2] BeckATSteerRABrownGK: Manual for the Beck Depression Inventory-II. *Psychological Corporation*.1996;

[3] van den NoortMPerriardBLimSBoschP: Why are self-rating results in patients with schizophrenia often unreliable?. *Psychiatry Res*.2017;251: 10.1016/j.psychres.2017.02.009 76-77 10.1016/j.psychres.2017.02.009 28189940

[4] NaberDMoritzSLambertMRajonkFHolzbachRMassRAndresenBFrankPRüdigerHReinhardMBurghardA: Improvement of schizophrenic patients’ subjective well-being under atypical antipsychotic drugs. *Schizophrenia Research*.2001;50(1-2) : 10.1016/S0920-9964(00)00166-3 79-88 10.1016/S0920-9964(00)00166-3 11378316

[5] HuangCYLiaoHYChangSH: Social desirability and the clinical self-report inventory: methodological reconsideration. *J Clin Psychol*.1998;54(4) :517-28 962375610.1002/(sici)1097-4679(199806)54:4<517::aid-jclp13>3.0.co;2-i

[6] ParienteJWhitePFrackowiakRSLewithG: Expectancy and belief modulate the neuronal substrates of pain treated by acupuncture. *Neuroimage*.2005;25(4) : 10.1016/j.neuroimage.2005.01.016 1161-7 10.1016/j.neuroimage.2005.01.016 15850733

[7] DhondRPYehCParkKKettnerNNapadowV: Acupuncture modulates resting state connectivity in default and sensorimotor brain networks. *Pain*.2008;136(3) : 10.1016/j.pain.2008.01.011 407-18 10.1016/j.pain.2008.01.011 18337009PMC2440647

[8] HuiKKNixonEEVangelMGLiuJMarinaONapadowVHodgeSMRosenBRMakrisNKennedyDN: Characterization of the. *BMC Complement Altern Med*.2007;7: 10.1186/1472-6882-7-33 33 10.1186/1472-6882-7-33 17973984PMC2200650

[9] YeoSvan den NoortMBoschPLimS: Ipsilateral Putamen and Insula Activation by Both Left and Right GB34 Acupuncture Stimulation: An fMRI Study on Healthy Participants. *Evid Based Complement Alternat Med*.2016;2016: 10.1155/2016/4173185 4173185 10.1155/2016/4173185 28053642PMC5178348

[10] UrryHLNitschkeJBDolskiIJacksonDCDaltonKMMuellerCJRosenkranzMARyffCDSingerBHDavidsonRJ: Making a life worth living: neural correlates of well-being. *Psychol Sci*.2004;15(6) : 10.1111/j.0956-7976.2004.00686.x 367-72 10.1111/j.0956-7976.2004.00686.x 15147488

[11] ShimamuraAP: Bridging Psychological and Biological Science: The Good, Bad, and Ugly. *Perspect Psychol Sci*.2010;5(6) : 10.1177/1745691610388781 772-5 10.1177/1745691610388781 26161890

[12] PenroseR: Fashion, faith, and fantasy in the new physics of the universe. *Princeton: Princeton University Press*.2016;

[13] Van den NoortMLimSBoschP: On the need to unify neuroscience and physics. *Neuroimmunology and Neuroinflammation*.2016;3(12) : 10.20517/2347-8659.2016.55 10.20517/2347-8659.2016.55

[14] KleinSB: What memory is. *Wiley Interdiscip Rev Cogn Sci*.6(1) : 10.1002/wcs.1333 1-38 10.1002/wcs.1333 26262926

[15] KleinS: Lost feeling of ownership of one’s mental states: the importance of situating patient R.B.’s pathology in the context of contemporary theory and empiricism. *Philosophical Psychology*.2016;29(4) : 10.1080/09515089.2015.1126815 490-493 10.1080/09515089.2015.1126815

[16] KleinS: A defense of experiential realism: The need to take phenomenological reality on its own terms in the study of the mind. *Psychology of Consciousness: Theory, Research, and Practice*.2015;2(1) : 10.1037/cns0000036 41-56 10.1037/cns0000036

# Response

**Published:** 2017-04-30

**Authors:** Patrizio Tressoldi

**Affiliations:** Dipartimento di Psicologia Generale, Università di Padova, Italy

Thank you for your accurate and constructive review and sorry for the multiple typos.

In the following we try to answer to all your main comments.

- …self-rating instruments, such as the Beck Depression Inventory-II (BDI-II)2, have their own limitations… how should psychologists/psychiatrists deal with those methodological limitations in daily clinical practice according to the authors? In our opinion, relying on those (structured) questionnaire outcomes only, does not seem the way to go.

 

**Reply: we acknowledged the limitations of all instruments and procedures for a complete assessment of 1PAs expanding the paragraph “First-person accounts are not always reliable” now moved before the Discussion. However, these limitations cannot be offset by 3PAs, but only improving the instruments and procedures for the knowledge of 1PAs, see for example Pastore M, Nucci M, Bobbio A and Lombardi L (2017). Empirical scenarios of fake data analysis: The Sample Generation by Replacement (SGR) approach. Front. Psychol. 8:482. doi: 10.3389/fpsyg.2017.00482; Lange R. Rasch scaling and cumulative theory-building in consciousness research. Psychology of Consciousness: Theory, Research, and Practice. 2017 Mar;4(1):135.**

 

- .. in the years after the Pariente et al. (2005) publication, the measurement of deqi scores of the participants in acupuncture studies were introduced and are now being collected alongside the 3PAs, consisting of the following Deqi sensations: aching, soreness, numbness, fullness, sharp or dull pain, pressure, heaviness, warmth, coolness, tingling, itching, and any others.

**Reply: in the “Pain” paragraph we added the procedure used by Hui et al. (2007) for the assessment of Deqi sensations. Their procedure confirms that these sensations can only be investigated by referring to only 1PAs and not 3PAs**

 

- To date, the studies on neural and psychophysiological correlates not at all contribute significantly, taking into account the large amounts of research funding/resources that have been invested so far. However, why do the authors think that it would be technically, hypothetically, impossible to combine 1PAs with 3PAs?.

**Reply: throughout our paper we presented examples where 1PAs and 3PAs are investigated together. However, our main thesis is that they offer very different information and that 1PAs cannot be obtained from 3PAs and hence are primary and irreducible.**

 

- ... we are wondering what the opinion of the authors is with respect to their claims, in terms of the fundamental laws of physics? Note that to date, a unified brain processing theory (unifying physics and neuroscience) does not exist. How do the authors think that a better theory of its underlying fundamental laws of physics could describe and explain 1PAs and 3PAs?

**Reply: We agree completely with the necessity to consider valid alternatives to the mainstream physicalism metaphysics as we pointed out in the Discussion. Such new alternatives must not only unify the fundamental laws of physics, that are not those of classic physics, with neuroscience but also with subjective qualia.**

 

- Finally, there are several minor issues that we would like for the authors to address in their final version of the paper.

**Reply: thank you very much for all these issues we fixed in the version 2 of the paper.**
